# Health inequities in environmental justice communities: relevant indicators to reflect a variety of health threats

**DOI:** 10.1186/1475-9276-11-S1-A7

**Published:** 2012-01-23

**Authors:** John Prochaska, Hilton Kelley, Stephen Linder, Ken Sexton, John Sullivan, Lexi Bambas Nolen

**Affiliations:** 1 Center to Eliminate Health Disparities, University of Texas Medical Branch, Galveston, Texas, 77555, USA; 2 Community In-Power Development Association, Port Arthur, Texas, 77642, USA; 3 Institute for Health Policy, School of Public Health, University of Texas, Houston, Texas, 77225, USA; 4 Division of Epidemiology, Human Genetics, and Environmental Sciences, School of Public Health, University of Texas, Brownsville, Texas, 78520, USA; 5 NIEHS Center in Environmental Toxicology, University of Texas Medical Branch, Galveston, Texas, 77555, USA

## Background

Residents of environmental justice (EJ) communities often suffer significant health inequities due to pollutants and to adverse social conditions. However, methodologies for assessing such communities seldom account for both kinds of factors. While traditional environmental health risk assessments use single-pollutant, single-source measures of chronic risk alone, this project develops a technique for assessing “overall risk burden” in EJ communities. Cumulative risks from aggregate (that is, multi-agent, multi-pathway, multi-source, over time) exposures are combined with an index representing a wide range of social determinants of health. The work is being piloted in West Port Arthur, Texas, an EJ community flanked by petrochemical plants and a seaport, and characterized by poverty and disadvantage.

## Materials and methods

Place-based social determinant indicators relevant to EJ communities were identified from Health Impact Assessment tools, then added to novel measures of cumulative risk developed by the U.S. Environmental Protection Agency and others, forming a composite index for overall risk burden [1,2]. Indicators were mapped to show spatial disparities across the community [3]. A community participatory mapping exercise was conducted to collect local knowledge of overall risk burden and to support local capacity to participate in and understand the project.

## Results

Preliminary findings suggest that the neighborhood’s proximity to petrochemical plants and to the seaport poses health risks to residents not only due to exposure to pollutants but also to the adverse health impact of living in an area with physical isolation due to poorly designed roadways, poor food security, poor child care availability, high unemployment and a depressed economy. Spatial concentrations of poverty and segregation further undermine health in the community. In fact, non-chemical social determinants of health may have a greater combined effect on health than exposure to toxicants in this community. Further study of this differential effect is needed.

## Conclusions

A technique of combined-indicator assessment provides a more complete understanding of the overall risk burden, the creation of health inequities, and the need for multiple intervention strategies to reduce health inequities. EJ communities should expand their focus to include social determinants of health. Strong partnerships between researchers and community residents are critical to: informing the selection of appropriate indicators, interpreting findings, and identifying action strategies. The work has important implications for how EJ communities identify priority issues, conduct research, and frame advocacy issues. Next steps include refining the assessment tool and testing the technique in other EJ communities.

**Figure 1:**
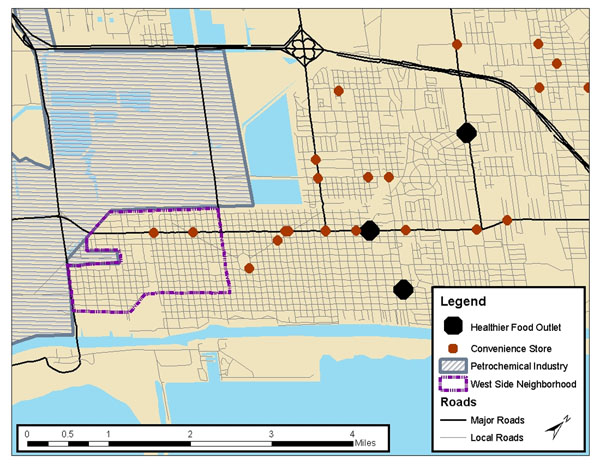
Access to Healthy and Unhealthy Food Outlets as an Example of Social Determinants of Health in West Port Arthur, Texas, 2011.

## References

[B1] United States Environmental Protection Agency. National-Scale Air Toxics Assessment, 2005 http://www.epa.gov/ttn/atw/nata2005 (accessed 10/20/2011)

[B2] San Francisco Department of Public Health. The Healthy Development Measurement Tool http://www.thehdmt.org (accessed 10/20/2011)

[B3] ESRI ArcGIS 9.3 Redlands, CA

